# Genes, Cognition, and Communication: Insights from Neurodevelopmental Disorders

**DOI:** 10.1111/j.1749-6632.2009.04419.x

**Published:** 2009-03

**Authors:** DVM Bishop

**Affiliations:** Department of Experimental Psychology, University of OxfordOxford, United Kingdom

**Keywords:** genes, language, communication, specific language impairment (SLI), dyslexia, autism, microcephalin, FOXP2, ASPM, copy number variants, single nucleotide polymorphisms (SNPs), copy number variant (CNV)

## Abstract

Twin and family studies have demonstrated that most cognitive traits are moderately to highly heritable. Neurodevelopmental disorders such as dyslexia, autism, and specific language impairment (SLI) also show strong genetic influence. Nevertheless, it has proved difficult for researchers to identify genes that would explain substantial amounts of variance in cognitive traits or disorders. Although this observation may seem paradoxical, it fits with a multifactorial model of how complex human traits are influenced by numerous genes that interact with one another, and with the environment, to produce a specific phenotype. Such a model can also explain why genetic influences on cognition have not vanished in the course of human evolution. Recent linkage and association studies of SLI and dyslexia are reviewed to illustrate these points. The role of nonheritable genetic mutations (sporadic copy number variants) in causing autism is also discussed. Finally, research on phenotypic correlates of allelic variation in the genes *ASPM* and *microcephalin* is considered; initial interest in these as genes for brain size or intelligence has been dampened by a failure to find phenotypic differences in people with different versions of these genes. There is a current vogue for investigators to include measures of allelic variants in studies of cognition and cognitive disorders. It is important to be aware that the effect sizes associated with these variants are typically small and hard to detect without extremely large sample sizes.

Most human genes have the same DNA sequence, for all people. If a gene takes the same form in virtually everyone it is said to be “fixed” in the population. Although mutations of fixed genes sometimes occur, when they do they are often either lethal or associated with disease or disability. When common individual differences among people are shown to be heritable, this points to a causal role for genes that are not fixed, but that show allelic variation from person to person.

Studies of twins provide a means of teasing apart genetic and environmental influences on a trait and have demonstrated moderate to high heritability for a host of cognitive traits and disorders, including verbal and nonverbal intelligence (Bouchard 1998), language skill (Stromswold 2001), reading ability (Harlaar et al. 2005), specific language impairment (SLI) (Bishop 2002), dyslexia (Grigorenko 2004), and autism (Rutter 2005). Such findings led researchers to expect that once we developed sufficiently sensitive molecular genetic tools we would readily identify genes that were implicated in cognition generally and communication more specifically. Yet, after more than a decade of research toward this goal, it is clear that the task is far harder than anyone anticipated. One way of reconciling the moderate-to-high heritability of cognitive abilities with the lack of genes of large effect is to postulate that there are common allelic variants that affect cognitive function, but they are individually of small effect size and do not have their effects in isolation: They operate against a background of genetic influences and their impact may also be affected by environmental factors. Thus, an allelic variant may be associated with lowered ability only when it co-occurs with disadvantageous alleles on other genes or with specific environmental circumstances; against a different background, it may be neutral, or even advantageous.

Research conducted over the past decade has increasingly supported a multifactorial account of genetic influences on individual differences in human cognition, but when researchers consider specific neurodevelopmental disorders, there is less agreement. According to one view, the etiology of disorders is no different from the etiology of individual differences in the normal range: Both are affected by the combined influence of many common variants, the effects of which are small, and interdependent with one another and the environment (Plomin & Kovas 2005). However, an alternative possibility is that we are dealing with numerous but heterogeneous genetic variants that are individually very rare (Keller & Miller 2006). Thus, there might be genes that have a large effect size in affected individuals, but only a small effect at the level of the population, because they affect only a tiny minority of cases. Rather than attempting to review the enormous and growing literature on genetic influences on normal and impaired cognition, I shall use examples from neurodevelopmental disorders to illustrate these issues before briefly reviewing studies on the role of copy number variants in autism, as well as on microcephalin and ASPM, two putative genes for brain size. Finally, I shall consider how we would integrate what we know about genetic influences on human cognition and communication with evolutionary considerations.

## Language Development and Disorders

Language is found in all human cultures. Regardless of whether they grow up in a Western city or a remote Amazonian forest, children learn to talk. Furthermore, this remarkable skill, unlike anything seen in another species, is mastered within around 4 years. Languages vary substantially in the sounds and rules they use to convey meaning, and we are still a long way from understanding how language learning is achieved, but it is clear that most children acquire it rapidly and without explicit instruction. Furthermore, language learning is remarkably robust in the face of neurobiological insults, such as perinatal brain damage, and environmental adversities, such as limited language input (Bishop & Mogford 1988). Even severe hearing loss need not handicap language acquisition, provided the child is exposed to a sign language (Neville & Mills 1997). Nevertheless, some children have problems with language acquisition for no apparent reason. In most cases, these children learn to talk, but they master language milestones much later than normal, and they may continue to use simplified syntax and vocabulary into adulthood. Where such a picture is seen in a child of otherwise normal intelligence for no apparent cause this is termed *specific language impairment* (SLI). Although it is less well-known than developmental dyslexia or autism, SLI is a relatively common developmental disorder. The boundary between SLI and normality is not clear cut and prevalence rates depend on how it is defined: Tomblin et al. (1997) gave estimates of 3 to 7%, depending on the cutoffs used.

For many years it was assumed that SLI was caused by inadequate parental communication, but a trio of twin studies in the 1990s provided evidence of high heritability (see Bishop 2002 for review). One expects twins growing up together to resemble each other because they are subject to the same environmental influences. If, however, monozygotic (MZ) twins, who are genetically identical, are more concordant for disorder than dizygotic (DZ) twins, who share on average only half their segregating genes, this is evidence for a genetic influence on disorder. Findings from twin and family studies suggested that it would only be a matter of time before a gene for SLI would be discovered. The expectation seemed fulfilled when a mutation of the FOXP2 gene was found to co-segregate perfectly with speech and language disorder in a three-generational British family, the KE family (see Fisher 2005, for review). Much debate followed over whether this was a “gene for language” or even a “gene for grammar,” an oversimplistic and sensationalist view that the researchers were at pains to dispel (Fisher 2006). Comparative studies indicated that this gene was highly conserved (similar in DNA sequence) across mammalian species, with only three amino acid substitutions distinguishing between FOXP2 proteins in a human and a mouse. Two of these changes had occurred after divergence of the lineage between chimpanzees and humans, and further analyses of the gene identified evidence that high survival value had led to the changes rapidly spreading through human populations. As Fisher (2006) noted, the gene is a transcription factor that regulates other genes and had impact on many systems, not just the brain; nevertheless, it is clear that disruption of its function affects development of brain regions important for language. However, FOXP2 is not a general explanation for SLI; investigations of other affected individuals seldom found any mutations of this gene (Newbury et al. 2002), except in a handful of cases with a similar complex phenotype involving both verbal dyspraxia and language deficits (Macdermot et al. 2005).

Other researchers conducting twin studies of SLI argued that in the majority of cases it is not a distinct disorder, but rather the extreme end of a normal distribution of language ability, likely to be influenced by multiple genetic and environmental influences of small effect (Plomin & Kovas 2005). Similar conclusions have been reached about a host of physical disorders, such as heart disease, diabetes, and allergies, with geneticists talking of complex multifactorial disorders (Sing & Reilly 1993). There were several reasons for this shift in conceptualization. First, many common disorders, including SLI, do not usually show family pedigrees indicative of classic Mendelian inheritance: As Sing and Reilly succinctly put it, these disorders aggregate but do not segregate in families. In this regard, the KE family is the exception rather than the rule, with 15 family members across three generations showing an autosomal dominant pattern of inheritance (i.e., the probability of an affected parent having an affected child is 50%). A second reason for favoring a multifactorial model is when a disorder is common. As argued by Keller and Miller (2006), most Mendelian disorders that affect reproductive fitness have very low prevalence because of selectional pressures against the mutation. If we accept that language proficiency would have conferred a reproductive advantage for ancestral humans (Pinker 2003), then it is hard to explain the persistence of common heritable language impairments in terms of a single gene of large effect. A third line of evidence concerns the relationship between disorder and the distribution of abilities in the population as a whole. SLI, like many other disorders, does not have pathognomic features; rather, it is defined in terms of arbitrary cutoffs on a continuum of language ability. Plomin and Kovas (2005) added a more technical argument, one based on a specific analytic method that gives an estimate of “group heritability”—the heritability for extreme scores on a dimension. They argued that if group heritability is significant, then this is evidence of genetic continuity with normality. However, neither of these latter two lines of evidence is watertight. We know that the KE family's problems are caused by a very rare mutation, yet their difficulties can still be quantified in terms of low language test scores. More generally, there are some features of SLI that are not normally distributed in the population; rather most children acquire full competence by around 4 years of age, leaving a tail of cases with persisting difficulties. Apparent continuity with normal-range performance may simply be a consequence of the measuring scale. This is the case for problems using verb inflections (Bishop 2005) and difficulties in speech production (Bishop & Hayiou-Thomas 2008), both of which are highly heritable. Using simulations, Bishop (2005) showed not only that significant group heritability could be found for a disorder caused by a rare mutation, but that the pattern of results for verb inflection difficulties was more consistent with such a cause, rather than that of multifactorial inheritance. Thus, it seems likely that rare variants could account for at least some cases of SLI other than those in the KE family.

Nevertheless, it seems probable that for many common forms of SLI we are unlikely to find individual genes of large effect; rather the etiology will be complex and involve a constellation of influences, each of which is small in magnitude. An important question for future research is whether we can distinguish phenotypically between forms of SLI that are caused by rare genetic variants, those that are heritable but with complex polygenic etiology, and those that are more environmental in origin. To resolve such questions we may need to move from traditional clinical methods of assessment and diagnosis and focus instead on theoretically motivated measures of underlying processes of perception, memory, and linguistic representations (Bishop 2008).

How, then, are we to discover the relevant genes? The main approach available to molecular geneticists doing the first studies in this field was linkage analysis, which involves looking for genetic markers that are shared at above chance frequency in affected individuals from the same family (see Newbury & Monaco 2008 for overview). This method relies on the fact that there are many regions of the genome that show a high degree of variation from one individual to another. Often these are in noncoding regions, thought to be unimportant for causing individual differences. However, their variability makes them useful because it is unlikely that any two unrelated people will have the same DNA sequence—this means that one can track how the DNA sequence in a given chromosome region relates to the phenotype in multiple people from the same family. A common misconception is that discovery of linkage equates to identification of genes that cause disorder. In fact, the highly variable genetic markers used in linkage analysis are unlikely to be functional. However, sections of DNA that are close together tend to be inherited together in blocks, so if we find significant linkage to a DNA marker then there is a good chance that a gene close to that marker may be involved. Thus, genetic markers act as signposts to regions of the genome that are likely to harbor risk genes. The identified region may contain many different genes, and further painstaking work is needed to characterize all of these and look for mutant DNA sequences within them. To illustrate this point Newbury and Monaco (2008) noted that the linkage region initially identified in the KE family contained around 100 genes, many of which were plausible candidates because they were known to affect neurological function. Progress in identifying FOXP2 might have been much slower, had it not been for a fortuitous discovery of a single case with a similar phenotype having a chromosomal translocation that disrupted this gene.

Linkage analysis is a useful method when looking for genes of major effect, especially if the same genes are involved in different families. It is less good at detecting genes of small probabilistic effect, although the method can be made more powerful when quantitative traits are considered, where one looks for a relationship between degree of genetic similarity and degree of phenotypic similarity between individuals in a pair. Increasingly, with the advent of fast automated genetic analysis, researchers are moving to an alternative method, association analysis. Association analysis is conceptually rather simpler than linkage analysis in that it involves categorizing individuals in terms of allelic status at a given locus, and looking for associations with phenotypic measures. It is much more sensitive than linkage analysis to small effects; however, the sensitivity of association analysis is counteracted by the fact that it is effective only if the marker is very close to the actual risk gene. Association analysis has traditionally been used to home in on regions that were identified by linkage analysis or to test for association with specific genes that were strong candidates because their function was known. It has now become more common in molecular genetic studies to perform association analysis covering the whole genome by considering associations with a large array of single nucleotide polymorphisms (SNPs) (i.e., variations at a single nucleotide site that differ between members of a species; McCarthy et al. 2008). However, the simplicity of this approach is deceptive, because on the one hand the associations are likely to be weak and probabilistic, and on the other hand the number of loci that are screened is potentially enormous—in current studies typically running into the hundreds of thousands. This poses problems of interpretation when an association is found, because adjustment has to be made to *p*-values to take into account the inflated probability of chance findings. But, if such correction is too stringent, one may end up dismissing associations that are small but genuine.

One solution is to adopt a two-stage procedure in which the first genome scan is used to identify target markers that look promising, and the second to replicate in a new sample (Thomas et al. 2005). However, since each sample requires large numbers to detect weak associations, such work is time-consuming and expensive. Furthermore, failures to replicate still occur even when a statistically conservative multistage approach is used. Another way to improve reliability of findings is to capitalize on the fact that contiguous SNPs tend to be inherited together in a “haplotype block” and to look for associations between phenotypes and constellation of alleles in a block (Daly et al. 2001). An analogy of the difference between doing association analysis using SNPs and using haplotypes would be passing through a city on a train and noting just one letter of the station name: doing this give the rider a clue as to location, but a sequence of four consecutive letters is far more informative as to where one is. Similarly, haplotype analysis is more likely than analysis of single SNPs to generate replicable findings. Ultimately, however, it has to be remembered that the genes of interest do not directly code for behavior: They determine which proteins are produced by cells, thereby influencing brain structure and function. Proof of a causal role for a gene requires studies of its mode of action, with a demonstration that allelic variants affect expression levels of proteins that serve key functions in the neurobiology of the trait in question (Newbury & Monaco 2008). Until this is done we cannot know whether a SNP or haplotype that is associated with a phenotype is a functional variant or merely a nearby marker.

Molecular genetic studies of SLI have been conducted by the SLI Consortium (SLIC), who assembled a large group of families affected by SLI from both epidemiological and clinical samples. They focused on three main measures of the phenotype: (1) scores from expressive and (2) receptive composites of a widely used clinical language assessment, and (3) a test of nonword repetition, previously shown to demonstrate particularly high heritability in twin studies by Bishop et al. (1996, 2006), and regarded as a measure of phonological short-term memory. Linkage was found between a region on the long arm (q) of chromosome 16 and the nonword repetition phenotype, and between a region on the short arm (p) of chromosome 19 and the expressive language score (SLI Consortium 2002). Both linkages have been replicated in additional samples, though the specific language traits linked to chromosome 19 are not consistent from study to study (Falcaro et al. 2008; SLI Consortium 2004). These two linkage sites were not, however, significant in studies by a North American group, who instead reported significant linkage to chromosome 13 (Bartlett et al. 2002). Lack of agreement in results of genome scans is an all-too-common finding and raises concerns about false positives, despite statistical attempts to control for these. However, other explanations are plausible: Results can be influenced by different methods of sampling, of phenotypic measurement, or choice of statistical method. Furthermore, where there is genuine but weak linkage, random sampling error will affect whether it is detected.

Our ability to home in on genes relevant to SLI is hampered by our limited understanding of how genes build a brain that can learn language. Vernes et al. (2008) adopted a novel approach by taking as a starting point the findings from the FOXP2 gene, which is known to have a role in switching on and off other target genes. Although mutations of FOXP2 are causal in only a small minority of cases of SLI, these authors reasoned that FOXP2 regulates other genes that are important in the development of neural pathways implicated in language, and so they hypothesized that such genes might be involved in cases of typical SLI. Vernes et al. carried out a functional genomic screen for FOXP2 targets and discovered that FOXP2 binds to and downregulates a gene on the long arm of chromosome 7 called “contactin-associated protein-like 2” (CNTNAP2). CNTNAP2 is a polymorphic gene known to be important in neural development. Around the same time that Vernes et al. were conducting their study, Abrahams et al. (2007) had been looking for genes that show differential expression in the fetal brains of humans and rodents, and identified CNTNAP2 as one of two genes that showed strikingly higher and more focal expression in human prefrontal cortex compared to other regions, and generally higher cortical expression in humans than in rodents. Using the sample from the SLIC Consortium, Vernes et al. showed that in children with typical SLI, nonword repetition scores were significantly associated with polymorphisms of this gene. Using a cluster of nine SNPs that showed association with nonword repetition, the researchers identified four haplotypes that among them accounted for 94% of individuals. The most common of these haplotypes had a frequency of 35%, and was found in 40% of those with nonword repetition deficits (performance more than 2 SD below the population mean), as compared with 29% of those with good nonword repetition (more than 0.5 SD above the population mean). Children who had no copies of this haplotype had a mean standard score of 95.2 on nonword repetition, those carrying one copy had a mean score of 89.7, and those with two copies had a mean score of 89.4, consistent with a dominant effect. It is noteworthy that the effect size of this haplotype, at just below *d*= 0.4, is large relative to many of the associations described in this field (see below); nevertheless, presence of the risk haplotype does not guarantee poor nonword repetition—indeed the majority of those with poor nonword repetition scores did not have this risk haplotype, and many of those with good nonword repetition did have one or two copies of it. A further point to note is that replications of association studies are always important, and they frequently find smaller effect sizes than the original study. Also, the association does not seem specific to SLI: Genetic differences in CNTNAP2 have also been associated with autism (Alarcón et al. 2008) and schizophrenia (Friedman et al. 2008).

What can this tell us about genetic influences on normal language development? One conclusion is that language development is robust in the face of genetic as well as environmental risks; the fact that many people with risk alleles do not develop frank disorder suggests that it is unusual for a “single hit” to compromise language development. This conclusion from genetics is nicely consistent with behavioral data, suggesting that deficits associated with SLI, such as ones affecting memory or auditory perception, may be seen in family members who do not themselves show any severe language difficulties (Barry et al. 2007, 2008). This makes sense if one assumes that language shows strong “canalization” (Waddington 1942), so that a range of genotypes can produce the same phenotype. Only when there are two or more factors disrupting language processes will an overt deficit be observed (Bishop 2006). If this is the case then genetic studies might be most fruitful if they focus on component aspects of the phenotype, which run in families but are only probabilistically associated with clinical-level impairment.

## Reading and Developmental Dyslexia

Language impairment and reading disorders often go hand in hand, yet from a genetic perspective there is an important difference between them. Oral language is a universal human characteristic with obvious survival value. Written language, on the other hand, is a human invention that is not found in all societies and is of recent origin in the scale of human evolution. Although literacy has clear benefits in enabling acquisition of knowledge across, as well as within, generations, it is not clear that illiteracy affects reproductive fitness, and persistence of genetic variants that selectively impair reading would pose far less of a paradox than would persistence of variants affecting oral language.

The diagnosis of developmental dyslexia is made when a child has unusual difficulty learning to read for no apparent reason. The disorder is typically defined in terms of a substantial mismatch between general intelligence and literacy skills, although the logic of this approach has been challenged (Lyon 2003). A genetic basis for dyslexia was recognized in some of the earliest work on this topic (see Schumacher et al. 2007 for review), with Hallgren (1950) noting that dyslexia often ran in families and suggesting that genes were implicated. Subsequent twin studies have largely confirmed this impression; two large-scale two studies, the Colorado Twin Study (Wadsworth et al. 2007) and the Twins Early Development Study (Harlaar et al. 2005) both found significantly higher concordance for reading disability in MZ than in DZ twins.

Twin studies provide evidence that genes are implicated in developmental dyslexia, but they give no indication of how many genes are involved. As with SLI, when researchers first started to investigate the genetics of dyslexia there was an expectation that we might find a single dominant or recessive gene that could explain the disorder. However, this did not turn out to be the case. Rather, probabilistic linkages were reported. In the first linkage study conducted in this area, Smith et al. (1983) focused on a group of nine families in whom dyslexia appeared to be inherited in an autosomal dominant manner. They tested 21 markers and found linkage to a region of chromosome 15, with one family contributing substantially to this result. Although the precise location has varied from study to study, linkage to the long arm of chromosome 15 was subsequently replicated both in an extended study by Smith and colleagues, and by other groups (see Fisher & DeFries 2002 for review of the early work).

With the passage of time a wider range of markers and more sophisticated methods of analysis became available, allowing further investigations of the same families. Using a quantitative approach to linkage analysis, Cardon et al. (1994) found linkage to a region of the short arm of chromosome 6 (6p22.2). This was subsequently replicated in several independent samples, though there have also been some failures to replicate (see Fisher & DeFries 2002; Fisher & Francks 2006).

In the past few years, researchers have identified specific genes that appear to be implicated in dyslexia. Findings do not always replicate, and it can be hard to know whether this is because of type I error, lack of power to detect small effects, or heterogeneity between populations. I shall focus on just on one region on chromosome 6 where considerable progress has been made in the past few years, but for detailed critical review of other putative associations see Paracchini, Scerri, and Monaco (2007).

Using samples from the UK and US, Francks et al. (2004) refined the linkage region on 6p22.2 to a 77-kb region that spanned two genes, TTRAP and KIAA0319. Within this region they identified a risk haplotype, tagged by three SNPs, that had a frequency of around 12% in these samples and had an average effect between −0.23 and −0.34 SD (depending on the sample) on IQ-adjusted reading measures. Cope et al. (2005) focused on a region of chromosome 6p22.2 containing 7 candidate genes, evaluating 137 SNPs in this region in an independent UK sample. A multistage analysis was used, first identifying SNPs that showed association with dyslexia in pooled DNA samples, then evaluating the association in individual cases and controls, as well as looking at associations within families. In addition, haplotype analysis was conducted. Significant association was found for a two-SNP haplotype within the KIAA0319 gene: The most common form with alleles 1–1 was equally frequent for affected versus unaffected cases, but two other common forms, 1–2 and 2–1, showed contrasting effects. (Conventionally, 1 is the more frequent allele, and 2 is the less frequent). The 1–2 haplotype was found in 35% of those with dyslexia and 27% of those without, whereas for 2–1, the figures were 24% in those with dyslexia and 36% in unaffected controls. Note that while the report of this chapter is titled “Strong evidence that KIAA0319 is a susceptibility gene for developmental dyslexia,” this is not the same as saying that KIAA0319 conveys strong susceptibility. Extrapolating to the general population, one would expect that most individuals with the 1–2 risk haplotype would not be dyslexic, and most dyslexic individuals would not have the 1–2 haplotype. In a further analysis of the samples of both Francks et al. and Cope et al., Harold et al. (2006) analyzed all the markers previously identified by those researchers as well as additional SNPs in this region and found further evidence for association of dyslexia with KIAA0319, with five SNPs showing association in both samples. Interestingly, they found only weak and inconsistent support for association with another gene located close to KIAA0319, namely DCDC2, which had previously been reported as also associated with dyslexia.

Paracchini et al. (2006) noted that a causal role for variation in KIAA0319 in dyslexia would be supported if it could be shown that the risk haplotype affected neural function. They conducted studies in human cell lines using mass spectrometry to compare the level of transcription generated from chromosomes with high or low risk haplotypes. The experiment was carried out in different cell types (neuroblastoma and lymphoblastoids) using multiple genetic markers. Control cell lines were also tested to guard against type I error. The results indicated that the risk haplotype was associated with reduced gene expression of KIAA0319. This result requires replication, as it was based on just six individuals, but it fits well with data from Harold et al. (2006) indicating that the putative functional mutation is likely to reside within the regulatory region of KIAA0319. Paracchini et al. (2007) further reported that KIAA0319 affects neuronal migration, providing a plausible link to previous neuroanatomical work showing abnormal neuronal migration in the brains of those with dyslexia (Galaburda et al. 2006). They noted, however, a problem with this hypothesized causal route, which is that impairment of neuronal migration would be expected to have a broad impact on many aspects of cognitive development, rather than a selective effect on reading. Nevertheless, it is possible that a reading-specific vulnerability could be induced if the regional expression of the gene were moderated by the effect of other genes. Another puzzle, though, concerns the extent to which a mechanism of abnormal neuronal migration fits with a view of dyslexia as continuous with normality. Although one can undoubtedly have degrees of severity of migrational abnormalities, these abnormalities are usually regarded as a pathological phenomenon. The fact that association between haplotypes and dyslexia is most striking when severe phenotypes are used is consistent with results from a sample studied by Deffenbacher et al. (2004) and is another pointer to the possibility that at least some forms of dyslexia may be etiologically distinct from the low end of the normal range.

This contrasts with a multifactorial conceptualization, which regards dyslexia as being influenced by the same causal factors as operate across the whole continuum of reading ability. If this is correct, then it should be possible to identify relevant genes not only in rare dyslexic samples, but also in general population samples, where one would attempt to identify allelic variants that were predictive of reading across the range of ability. Two studies looked at haplotypes of KIAA0319 in general population samples. Luciano et al. (2007) used a sample of 440 adolescent twins and their parents who had been used to obtain estimates of genetic and environmental influences on literacy-related traits. Although they reported significant associations with 2 of 10 studied SNPs in this gene, and with a three-SNP haplotype, around half the associations were in the opposite direction to those previously reported in studies of dyslexic samples. This puzzling result could just represent type I error, but it is not the only case of a “flip-flop” phenomenon, whereby association with a risk allele is replicated, but in the opposite direction. Lin et al. (2007) conducted simulations to show that the extent to which different markers were co-inherited (i.e., in linkage disequilibrium) can vary from one population to another, and that where the phenotype depends on a specific constellation of allelic variants, then flip-flops between populations can occur. This further emphasizes the extent to which phenotypes depend on genetic constellations rather than individual genes. Another point to note is that Luciano et al. did not correct for IQ, and given that reading and IQ tend to be correlated, the phenotype they studied would be rather different from those in samples of dyslexics, who are usually identified on the basis of a mismatch between poor reading and average or high IQ. Given the very weak evidence for replication found by Luciano et al., it is of interest to find more positive results in a general population sample studied by Paracchini et al. (2008). They analyzed a set of SNPs and haplotypes that had previously been identified as risk or protective factors for dyslexia in over 5,000 children from a new population-based sample for whom reading measures were available. The three-SNP haplotype previously identified as a risk factor by Francks and colleagues again emerged as significantly associated with reading ability (and in the same direction), with the association improving when IQ was controlled. Depending on the specific measure used, the regression coefficients (which directly reflect change in z-score going from zero to one to two copies of the 1−1−2 haplotype) ranged from around −0.03 to −0.08. Though significant, this is considerably weaker than the association reported by Francks et al. They did not, furthermore, replicate the findings of Cope et al. for a two-SNP haplotype associated with good reading.

Developmental dyslexia is often presented as a success story for the field of genetics because specific linkages on chromosomes 6 and 15 have now been replicated across a number of samples. However, candidate gene associations account for only a small proportion of variance, in contrast to the high heritability estimates obtained from twin studies. This, of course, is just what would be predicted by a model of complex multifactorial etiology, but it emphasizes that we are not finding genetic variants that are necessary and sufficient for causing dyslexia. Take, for instance, the SNP rs2038137 in KIAA0319, which gave the most significant association in the chromosome 6p study of Harold et al. (2006) when both samples were considered together. The risk allele had a frequency of 70% in cases of dyslexia and 62% in controls in the Cardiff sample, a difference that would be far too small to be of use in predicting outcomes. Assuming a base rate of dyslexia of 10% in the population, in a sample of 1000 people, we would expect to find 628 with the risk allele, of whom 11% would have dyslexia, and 372 with a low-risk allele, of whom 8% would have dyslexia. Clearly, even where significant associations are replicated across several samples, the variants that have been found have only a small contributory effect to the etiology of dyslexia. There are two possible interpretations of this result: One possibility is that there is a functional variant that has a stronger causal relation with disorder, which is close to, but not identical with, the region identified by association analysis. But, another very plausible interpretation is that these risk factors correspond to genes of small effect and need to be combined with other genetic and/or environmental factors to exert their influence. When the first behavioral genetic studies of dyslexia revealed high heritability, many of us in the field anticipated that sooner or later there would be genetic tests that would make it possible to identify a child's risk of dyslexia before the start of schooling. The much more complex picture revealed by molecular genetic studies makes that goal seem increasingly unattainable.

## Autism and Copy Number Variants

When molecular geneticists first began to study autism, they anticipated that it would be relatively straightforward to find genes associated with this disorder, because all the phenotypic data indicated extremely high heritability (Barnby & Monaco 2003). However, despite a huge research effort from consortia using samples gathered from all over the world, progress has been slow. One possible reason could be that the phenotype is not appropriately specified. As we found for SLI, the clinical characterization of a disorder may not be optimal for defining a genetically meaningful phenotype. More progress may be made if autism is reconceptualized in a quantitative fashion, rather than as a syndrome. Furthermore, the possibility has been made mooted that different components of autism may have different genetic origins, with the full syndrome being observed only when a specific constellation of deficits is seen (Happé et al. 2006). Evidence came from Ronald et al. (2006), who gave a brief questionnaire regarding autistic-like symptoms to parents of a general population sample of twins and found that the three domains of social impairments, communication impairments, and restricted interests/repetitive behaviors showed only weak phenotypic correlations and little genetic overlap. Of course, finding that components of autism can fractionate is not strong evidence against a distinctive etiology for the syndrome: Consider, for instance, the case of Prader−Willi syndrome, caused by a deletion on chromosome 15 and characterized by excessive appetite, low muscle tone, and learning disabilities (Whittington & Holland 2004). If one were to do an analogous study to that of Ronald and colleagues in the general population, measuring these traits, it is unlikely they would have shared genetic variance, simply because Prader−Willi syndrome is a rare disorder that accounts for a tiny minority of cases. Similarly, the causes of the triad of impairments in autism could be unitary, even though they can fractionate in the general population. Stronger supportive evidence for Ronald et al.'s model comes from studies of relatives indicating that similar features, milder in kind and sometimes occurring in isolation, can be seen in cases of the “broader phenotype” (Dawson et al. 2002). Furthermore, the fact that the CNTNAP2 gene has been found to be associated with autism as well as with SLI (Alarcón et al. 2008) is consistent with the notion that there may be common genetic risk factors for both these disorders, which may be differentiated only in terms of there being other risk alleles in those with autism that lead to additional symptoms. It should be noted, however, that although this idea is currently popular, it is not fully supported by behavioral data on relatives; the broad phenotype of autism does not appear to encompass the kind of nonword repetition deficits associated with CNTNAP2 and seen in individuals with SLI and their relatives (Bishop et al. 2004; Whitehouse et al. 2007). The possibility of etiological overlap between SLI and autism is currently a focus of considerable research interest, but the jury is still out. One promising approach is the development of instruments that allow one to quantify underlying dimensions of autism; this allows us to assess subclinical features of this disorder in relatives in the quest for genotype−phenotype associations (Duvall et al. 2007).

One consequence of reconceptualizing autism as the result of a specific conjunction of “risk” alleles is that it might explain why such alleles persist in the population despite the fact that individuals with autism are unlikely to reproduce (Keller & Miller 2006). The argument is sometimes made that features of autism that are disadvantageous when they occur as part of a syndrome could be beneficial if they occur in isolation. For instance, Happé (1999) noted that the detail-focused cognitive style seen in autism could be advantageous under certain circumstances. Baron-Cohen (2000) made a similar case, quoting Temple Grandin, who herself has autism, as follows: “‘What would happen if you eliminated the autism genes from the gene pool? You would have a bunch of people standing around in a cave, chatting, and socializing and not getting anything done!’” (p. 491).

In the past few years, there has been growing interest in an alternative line of explanation for the failure to find genes for autism. The problem may have been not so much with phenotypic definition as with the nature of the molecular genetic investigations—investigations that have focused on looking for differences in genetic markers that typically encompass 1 to 10 base pairs. In addition to these conventional genetic markers, there is another kind of polymorphism, copy number variant (CNV), which operates on a much larger scale, involving deletions, insertions, duplications, and rearrangements of sections of DNA of length from 1000 base pairs up to several million base pairs (Beckmann et al. 2007; Redon et al. 2006). These may arise as spontaneous mutations, or be transmitted from parent to child. For years it was thought that gene dosage was determined solely by the alleles inherited from each parent, but it is now evident that dosage can also be affected by the presence of two copies of a gene on the same chromosome.

Two independent studies (Marshall et al. 2008; Sebat et al. 2007) reported increased rates of CNVs in individuals with autism. Intriguingly, most of the CNVs were not seen in the parents, indicating that they had arisen as sporadic mutations, rather than being inherited. It is ironic that the massive push for genetic studies of autism was prompted by twin and family studies that indicated high heritability, yet these latest results have revealed genetic anomalies that arose *de novo*. This raises questions as to whether the presence of CNVs may provide an explanatory mechanism only for a small subset of those with autism—as Beaudet (2007) noted, sporadic CNVs are associated with cases of autism that are atypical in that they are associated with other syndromic features and have an equal sex ratio. It will also be of interest to know how far such *de novo* mutations are related to paternal age, which has been linked with risk of autism (Reichenberg et al. 2006).

Another difficulty for studies of CNVs in relation to disorder is the fact that CNVs are so common in the general population. Beckmann et al. (2007) noted that in the HapMap project, when the focus was solely on differences at the level of the single nucleotide polymorphism, it was estimated that the difference between any two randomly selected genomes was only 0.1%, but this estimate has now been estimated upwards to at least 1%, with most of the difference due to CNVs. Because they can span regions of the chromosome containing many genes, CNVs can potentially affect a wide range of phenotypic characteristics. The problem, then, is that if one finds that an individual with autism has a CNV, it cannot necessarily be assumed that this is a factor in causing the autism (Abrahams & Geschwind 2008). It may be the case that research in this area will be less useful in identifying CNVs that cause disorder than in identifying genes that are duplicated or deleted by the CNV, which may be likely candidates for playing a role in autism.

## Microcephalin and Abnormal Spindle-Like, Microcephaly-Associated (ASPM)

The next example is something of a cautionary tale, showing how extrapolating from disorder to gene function in the general population is fraught with difficulties. The size and complexity of the brain is one of the most distinctive features differentiating humans from other primates (Passingham 2008). The brains of modern humans are more than 4 times larger than those of great apes. Genes that underlie this difference were discovered in the course of studying individuals affected by primary microcephaly. This condition is diagnosed when head circumference is at least 3 SD below the level expected for age and sex, in the absence of other syndromic features. Some forms of primary microcephaly are inherited as a recessive autosomal condition, and to date, six genes have been identified as important in the etiology (Cox et al. 2006). Typically, microcephaly is associated with mental retardation, often accompanied by other signs of neurological impairment such as motor handicap and seizures.

Microcephaly is a very rare condition, but its genetic basis aroused considerable interest when it was discovered that there is wide variation (polymorphism) in one of the genes that had been implicated, microcephalin, which when mutated leads to premature termination of synthesis of a protein involved in fetal brain development. Wang and Su (2004) documented this variation and compared human forms of microcephalin with those seen in 12 species of nonhuman primate. They found surprisingly high allelic variation in humans, whereas there was much less within-species variation in ape and monkey samples. If genetic diversity is due to selective pressure rather than random drift, then we expect to see more nucleotide replacements that alter a gene's protein product than replacements that do not. Subsequent work by Mekel-Bobrov et al. (2005) and Evans et al. (2005) produced such evidence for continuing adaptive evolution of both microcephalin and another microcephaly gene, ASPM (Abnormal Spindle-like, Microcephaly-associated), with both an ancestral form and a new derived form co-existing in human populations. For both genes, the data were consistent with positive selection pressure for the new derived form. Such findings fit with the idea that there were evolutionary pressures favoring the cognitive advantages of a large brain that outweighed the additional physiological costs of maintaining it. However, to confirm that microcephalin and/or ASPM were implicated in this development, one would need to show that there were indeed differences in brain size and cognition associated with the derived and ancestral forms of these two genes. Recent studies have failed to support these predictions. Woods et al. (2006) found no effect of genotype on brain volume measured using magnetic resonance imaging with 120 participants. Rushton et al. (2007) measured general mental ability, head circumference, and social intelligence in 644 Canadian adults of varied ethnic background and again found no relationship between these phenotypes and a person's genotype. The largest study conducted to date by Mekel-Bobrov et al. (2007) looked for association between the alleles of microcephalin and ASPM and intelligence in 2,393 individuals, and found no detectable effects of genotype on phenotype. These are sobering findings following the initial excitement regarding these two genes, and they emphasize that one cannot always predict what the correlates of common genetic polymorphisms will be from knowledge of the effects of a pathological mutation affecting the same genes. As Woods et al. (2006) noted, both microcephalin and ASPM are expressed in organs other than the brain, and it may be that positive selection for the derived variants of these genes has nothing to do with cognition.

A final line of evidence about the possible effect of microcephalin and ASPM is indirect: Dediu and Ladd (2007) noted that there were population differences in the frequency of derived and ancestral haplotypes for both microcephalin and ASPM. For both genes the new derived forms are relatively common in Europe, North Africa, and parts of Asia, but rarer in sub-Saharan Africa. In other populations, the frequencies of derived forms diverge for the two genes. The authors noticed a correlation between the type of language spoken in a population and the frequency of allelic forms, with tone languages being most common in populations where the ancestral alleles predominate, and nontone languages being most common in those populations where the derived allele is found with higher frequency. Dediu and Ladd emphasized that they were not proposing that genotype determined the type of language that could be learned: in general a human child can learn any natural language it is exposed to. However, they suggested that a genotype that facilitated pitch discrimination might have played a role in determining which acoustic cues became linguistically salient when a language developed. Although the concordance between population genotypes and type of language is intriguing, this work has to be seen as hypothesis-generating rather than definitive (Nettle 2007). Nevertheless, it gives clear predictions, not least of which is the idea that a person's genotype will determine their ability to learn to hear tone distinctions. Even more speculatively, we might predict that versions of these genes will relate to language proficiency in opposite ways, depending on whether the language learned is a tone language. As yet these remain hypotheses in need of formal test.

## Why Do Genetic Influences on Cognition and Communicative Skill Persist?

A puzzling question for evolutionary biology is why an optimal genotype has not reached fixation in the species. Suppose, for instance, that we have an allelic variant that is associated with good verbal skill. There is good reason to believe that possession of articulate and complex language conveys benefits on the individual, and would have an impact on survival value and reproductive success in ancestral humans (Hurford 1991). Possession of oral language makes it possible to transmit information over time and space, to form social bonds, and to contemplate future as well as past events. The fact that in the past few generations people have had access to birth control, altering the relationship between ability and reproductive success, is irrelevant here, because evolution operates over a much longer time-scale. According to standard evolutionary theory, early humans with a “high verbal” form of the gene would have left more offspring than those without, so that this optimal form gradually would become the dominant one, even if the selective advantage was relatively slight (Pinker 2003). How, then, can we explain the persistence of a “nonoptimal” version of the allele in the population?Keller and Miller (2006) addressed this question in the context of mental disorders, but their conclusions have relevance for individual differences in cognition. They noted that a common line of argument is in terms of “balancing selection,” whereby an allele is maintained in the population because disadvantageous effects are counterbalanced by advantageous traits. Most genes are pleiotropic, that is, have multiple effects, and so one can envisage a situation where an allele might be maintained because, for instance, it affects the balance of verbal and nonverbal abilities, rather than the absolute level of either skill. Accounts of autism that stress the advantages as well as the disadvantages of the “autistic cognitive style” could be regarded as instances of this theory. However, Keller and Miller (2006) are highly skeptical about this type of explanation because it only works if the balance between advantage and disadvantage of a trait is very close.

Another possibility is that evolution of genes for cognition is still underway. If these genes emerged relatively recently they could still be subject to natural selection. We considered this possibility in relation to microcephalin and ASPM, both of which show all the hallmarks of genes currently undergoing selection in human populations. However, as we saw, there was little evidence that they play a role in influencing cognitive abilities within the normal range. While it would be naïve to assume that evolution had stopped with the emergence of modern humans, there is as yet no evidence for genetic variants that are adaptively evolving and influencing cognitive abilities.

For common, heritable, psychiatric disorders, Keller and Miller argued in favor of a “common disease/rare variant” model (Wright et al. 2003). According to this model there is little consistency in genotype from one affected individual to the next, and adverse mutations affecting any given locus are rare. Keller and Miller noted that complex behaviors (which would extend to language, intelligence, and social behavior) involve integrating many complex pathways and so are potentially vulnerable to mutations at many loci. Although some geneticists find this model “too depressing to contemplate” (Keller & Miller, p. 402), it seems increasingly plausible for autism (Abrahams & Geschwind 2008), and examples such as the KE family indicate that it applies to at least some cases of specific language impairment.

A final model maintains that cognitive and communicative skills are affected by the combined effect of many genetic and environmental influences, and individual alleles are unlikely to account for substantial amounts of phenotypic variance, even if heritability is high. When the phenotype is a disorder, this becomes the “common disease/common variant” model (Wright et al. 2003), but advocates of this view argue that the term *disease* is inappropriate, because there are no clear dividing lines between normality and abnormality (Plomin 2000). Insofar as this model applies, it predicts that we are likely to discover more genetic variants that have small effects, that are common in the general population, and that affect skills in the normal range as well as in disorders. As we have seen, research on both language and literacy disorders has been moving toward adoption of this model for at least a proportion of cases.

## Future Directions

Over the past 25 years the role of genes in cognition has changed from being a minority interest to a hot topic. This trend has been largely driven by technological developments in the field of genetics that allow for rapid analysis of an individual's DNA. Nowadays anyone who has access to samples of DNA—from blood or cheek-scrapings or other tissues—can send this material to a commercial laboratory which will categorize individuals according to whatever aspect of the genotype the researcher requests. Over recent years the field has changed to reflect these developments. In the early days, research proposals focused on behavior and on defining better ways of measuring phenotypes and building cognitive models of underlying processes. With the advent of brain imaging, proposals started to include a structural and/or functional imaging component, with the hope that identifying the brain regions and/or networks underlying the disorder might bring more order into a chaotic field. Within the past decade genetics has been bolted on as the latest weapon in the attack on complexity. Thus, whereas in the past we assessed subtypes of reading disability or activation of the frontal lobes, now we now can categorize people according to allelic variants in the hope that this will reveal clearer patterns. So far, however, the result has not been greater clarity; on the contrary, as each new methodology is incorporated into the study of disorders, greater degrees of complexity are encountered. This is to be expected given what has emerged about the multifactorial nature of the influences on cognitive abilities and disabilities. We should not anticipate one-to-one relationships between allelic variants and phenotypes, be they traditional behavioral measures or neurobiological endophenotypes (Flint & Munafò 2007). The amount of variance that will be accounted for by variations in a single gene will usually be tiny and difficult to detect except in very large samples. Plomin et al. (2006) put the field in a sobering context by noting that in a whole genome scan for variants associated with intelligence, the largest effect size found was 0.1%, well below Cohen's (1992) cutoff for a “small” effect size (*d*= 0.2). Does this mean that the whole enterprise of molecular genetics of cognition and cognitive disorders should be abandoned?

My own view is less downbeat. Genetic studies can help unravel the complex etiology of cognition and disorders, provided a biologically informed approach is adopted. The shotgun application of a whole genome scan to look for associations between allelic variants and cognitive traits is one way forward, but may not necessarily be the best method to generate replicable findings. An alternative approach is to use a rare monogenic disorder or an animal model as an entry point, and, from an understanding of the biological pathways involved, identify other genes that are likely to be implicated, as was done by Vernes et al. (2008) in the case of developmental language disorders. Another important issue is the extent to which gene expression varies according to the environment. When the search for susceptibility genes is conducted using a design that includes relevant measures of the environment, this opens up the possibility of exploring gene−environment interaction (Rutter 2006). This may reveal genes that have little or no effect in one context but exert a more powerful influence in another.

At one level, the complexity of genetic influences on human traits may be a cause for celebration. Certainly, it makes research in this area challenging, but it also keeps at bay the looming specter of genetic selection for cognitive traits. In the first half of the 20th century concerns were frequently voiced that, as humans became able to control their fertility, the more intelligent would reproduce less, leading to a decline in overall intelligence in the population. This might indeed be expected if IQ were determined by a few genes of major effect, whose effects were independent of environment. However, although a negative association between intelligence and fertility has been reported in the US during the last century (e.g., Retherford & Sewell 1989), the average intelligence of the population has increased over the same period (Flynn 1984). This provides further evidence for the multiplicity and complexity of influences on human cognition.
